# Aortic Dissection Mimicry Under Extracorporeal Membrane Oxygenation (ECMO) After Cardiac Arrest: A Case Report of Emergency Imaging Dilemmas

**DOI:** 10.3390/reports9030262

**Published:** 2026-08-10

**Authors:** Yueh-Cheng Tu, Meng-Yu Wu, Giou-Teng Yiang, Yu-Long Chen

**Affiliations:** Department of Emergency Medicine, Taipei Tzu Chi Hospital, Buddhist Tzu Chi Medical Foundation, No. 289, Jianguo Rd., Xindian Dist., New Taipei City 231, Taiwan; loududy@gmail.com (Y.-C.T.); skyshangrila@gmail.com (M.-Y.W.); jtyang0201@mail.tcu.edu.tw (G.-T.Y.)

**Keywords:** cardiac arrest, extracorporeal cardiopulmonary resuscitation (ECPR), extracorporeal membrane oxygenation (ECMO), aortic dissection, image artifact, pitfall, hemodynamic

## Abstract

**Background and Clinical Significance:** Peripheral veno-arterial extracorporeal membrane oxygenation (VA-ECMO) substantially alters aortic flow dynamics, generating catastrophic false-positive pathology on standard imaging. We report a case of ECMO-induced artifacts mimicking a Stanford type A aortic dissection (TAAD), which led to an unnecessary exploratory sternotomy. **Case Presentation:** A 67-year-old man underwent extracorporeal cardiopulmonary resuscitation (ECPR) for a shockable out-of-hospital cardiac arrest. Post-resuscitation chest computed tomography angiography (CTA) and preoperative transesophageal echocardiography (TEE) demonstrated a prominent flap-like structure in the ascending aorta, prompting emergency sternotomy. Intraoperative exploration revealed no intimal tear. Subsequent evaluation confirmed an acute anterior myocardial infarction, managed with coronary intervention. Following a dismal neurological prognosis due to hypoxic encephalopathy, VA-ECMO was palliatively withdrawn on day 9, and the patient expired on day 19. The interaction between retrograde ECMO flow and varying levels of intrinsic cardiac function dictates the topology of flow disturbances. Absent native flow creates contrast layering within the aortic root, whereas preserved native flow creates a volatile downstream watershed zone. Based on these distinct phenotypes, we propose a novel conceptual framework for tailor-made imaging strategies titrated to native flow strength—such as temporary ECMO flow reduction for preserved native output, or circuit contrast injections for profound cardiac depression. **Conclusions:** ECMO-related artifacts present substantial diagnostic pitfalls. Clinicians should adopt a context-aware approach, integrating multi-modality imaging with hemodynamic status to implement individualized, physiologically guided imaging protocols.

## 1. Introduction and Clinical Significance

We present a complex case involving a 67-year-old man who was brought to our emergency department after an out-of-hospital cardiac arrest (OHCA) and subsequently received extracorporeal cardiopulmonary resuscitation (ECPR). In the emergency department, rapid post-resuscitation imaging frequently drives time-critical diagnostic and therapeutic decisions in patients undergoing ECPR. In this case, computed tomography angiography (CTA) of the chest and transesophageal echocardiography (TEE) both revealed findings initially suggestive of a Stanford type A aortic dissection (TAAD); however, these findings were later proven intraoperatively to represent an imaging artifact.

To the best of our knowledge, this is the first report describing a pseudo-aortic dissection manifested simultaneously on both CTA and TEE in a single patient. Ultimately, this case highlights the substantial diagnostic challenges posed by extracorporeal support and underscores that even with a multimodality imaging strategy, clinicians must remain context-aware of native cardiac output, dynamically titrating imaging protocols and ECMO parameters to ensure diagnostic accuracy.

## 2. Case Presentation

A 67-year-old man with a history of well-controlled type 2 diabetes mellitus collapsed after experiencing acute chest pain. Emergency medical technicians found him in OHCA and immediately initiated cardiopulmonary resuscitation (CPR). The initial rhythm was pulseless ventricular tachycardia (VT). Given the presentation of a shockable OHCA rhythm, the emergency department ECPR team was promptly activated following pre-hospital notification (according to our institutional ECPR activation criteria; Supplementary Table S1).

**Figure 1:**
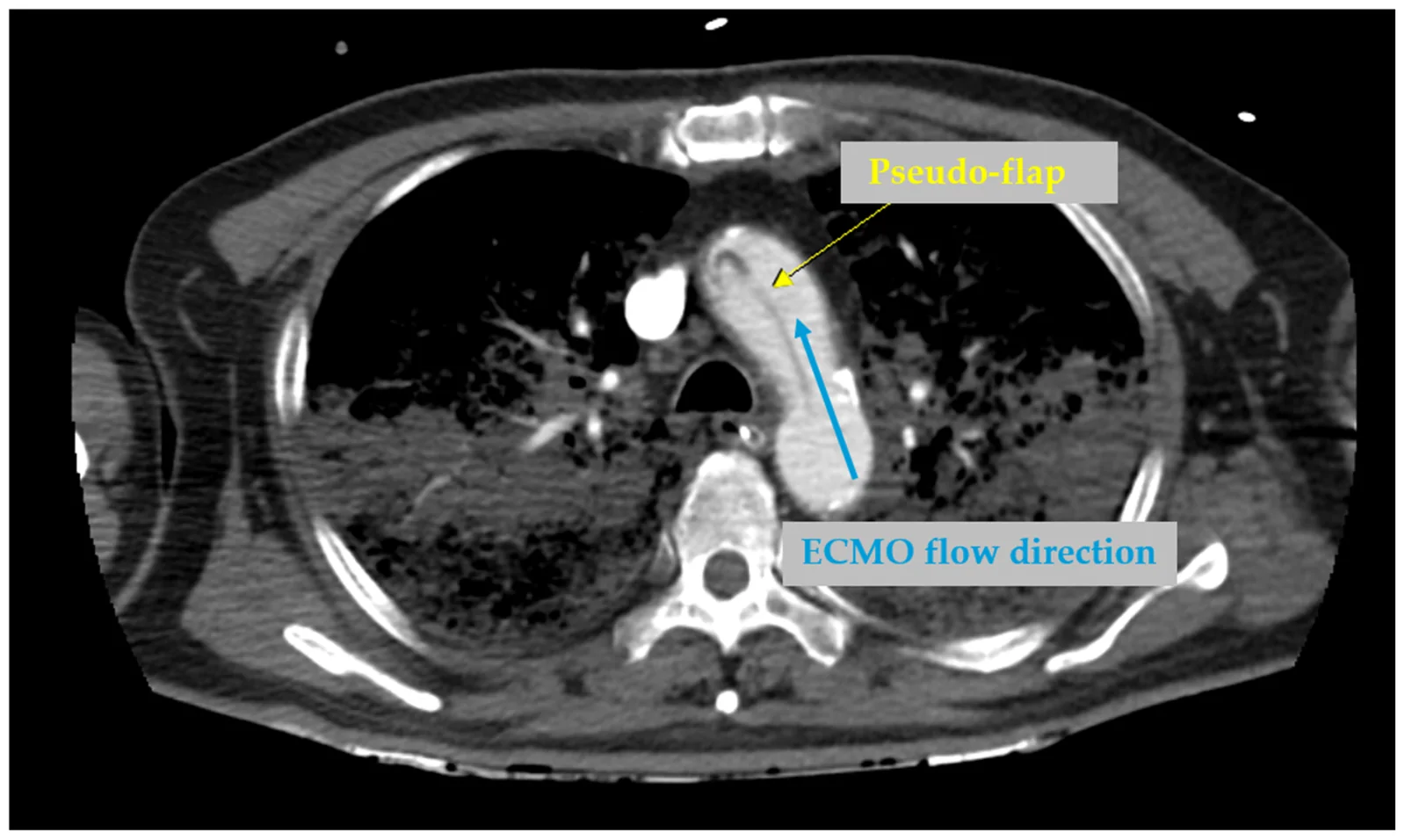
CTA artifact mimicking acute aortic dissection. Axial view reveals a pseudo-flap in the aortic arch (yellow arrow) generated by fluid dynamic interference. Retrograde, unenhanced flow from peripheral VA-ECMO (blue arrow) collides with enhanced, native cardiac ejection, producing a contrast-layering artifact indistinguishable from a true intimal flap.

Upon arrival at the emergency department, high-quality advanced cardiovascular life support (ACLS) was continued while peripheral veno-arterial ECMO was initiated simultaneously via femoral cannulation (15 Fr arterial and 21 Fr venous cannulas), with a pump speed of 6900 RPM and a flow rate of 3.0 L/min. Return of spontaneous circulation (ROSC) was successfully achieved after a total resuscitation duration of 42 min. Following ROSC, intra-arterial blood pressure was 120/88 mmHg without inotropic or vasopressor therapy. A bedside transthoracic echocardiography (TTE) revealed globally depressed ventricular wall motions, with an E-point septal separation (EPSS) >13 mm, indicating a severely reduced left ventricular ejection fraction (LVEF). However, the aortic root was poorly visualized, with no evidence of cardiac tamponade or aortic regurgitation. Subsequent chest CTA (dual-phase arterial chest CTA with contrast administration via a standard peripheral upper-extremity IV line; detailed scan timing and contrast injection protocol are provided in Supplementary Figure S1) appeared to demonstrate a type A aortic dissection with bilateral lung consolidation (Figure 1), prompting urgent surgical intervention.

At the time of CT acquisition, continuous quantitative native cardiac output and high-resolution arterial line pulsatility waveforms were not formally recorded due to the hyperacute nature of ECPR.

**Figure 2:**
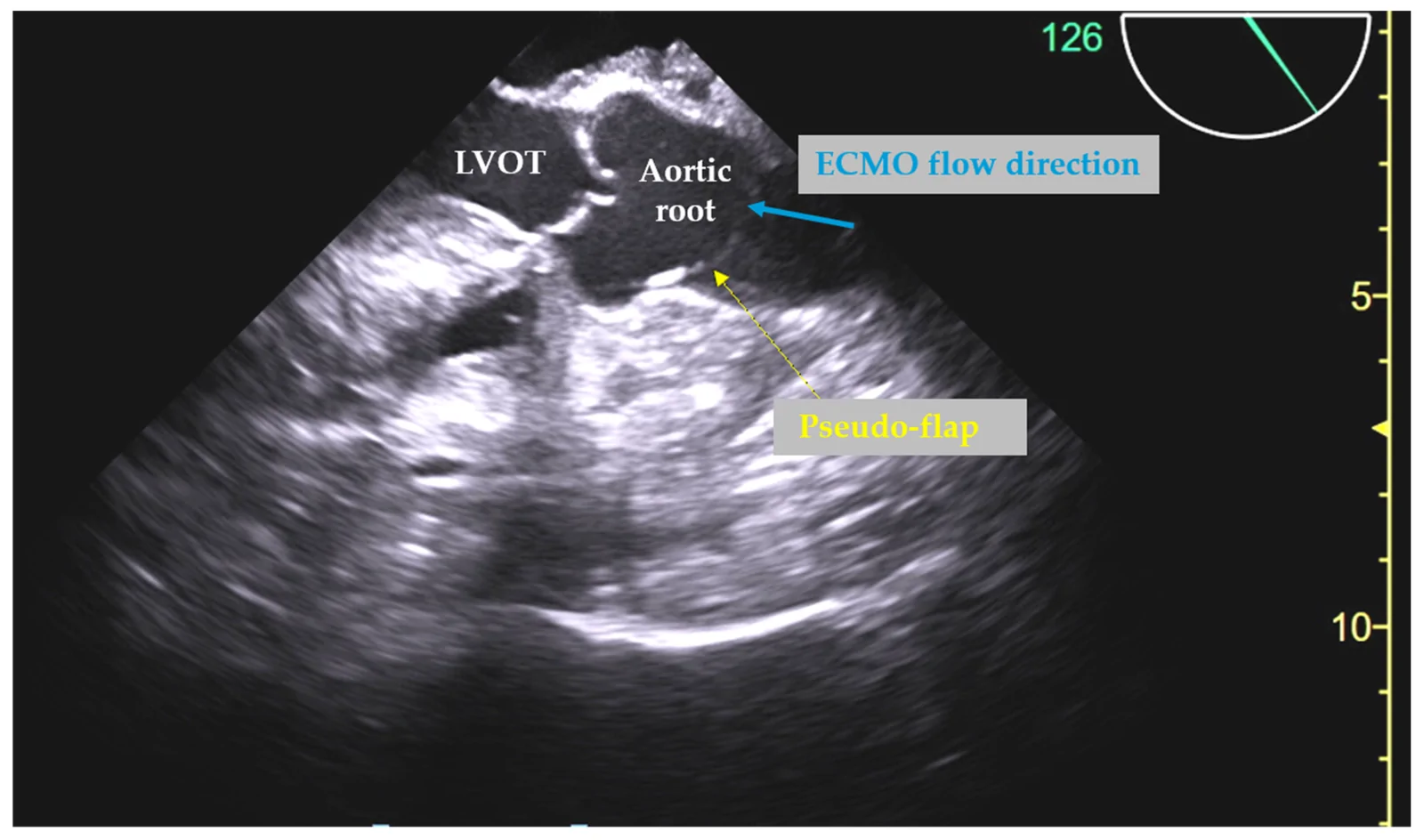
TEE artifact mimicking aortic dissection. Mid-esophageal long-axis view (126°) showing a false-positive intimal flap (yellow dashed arrow, pseudo-flap) within the aortic root, adjacent to the left ventricular outflow tract (LVOT). The retrograde peripheral VA-ECMO flow was demonstrated by the blue arrow.

**Figure 3:**
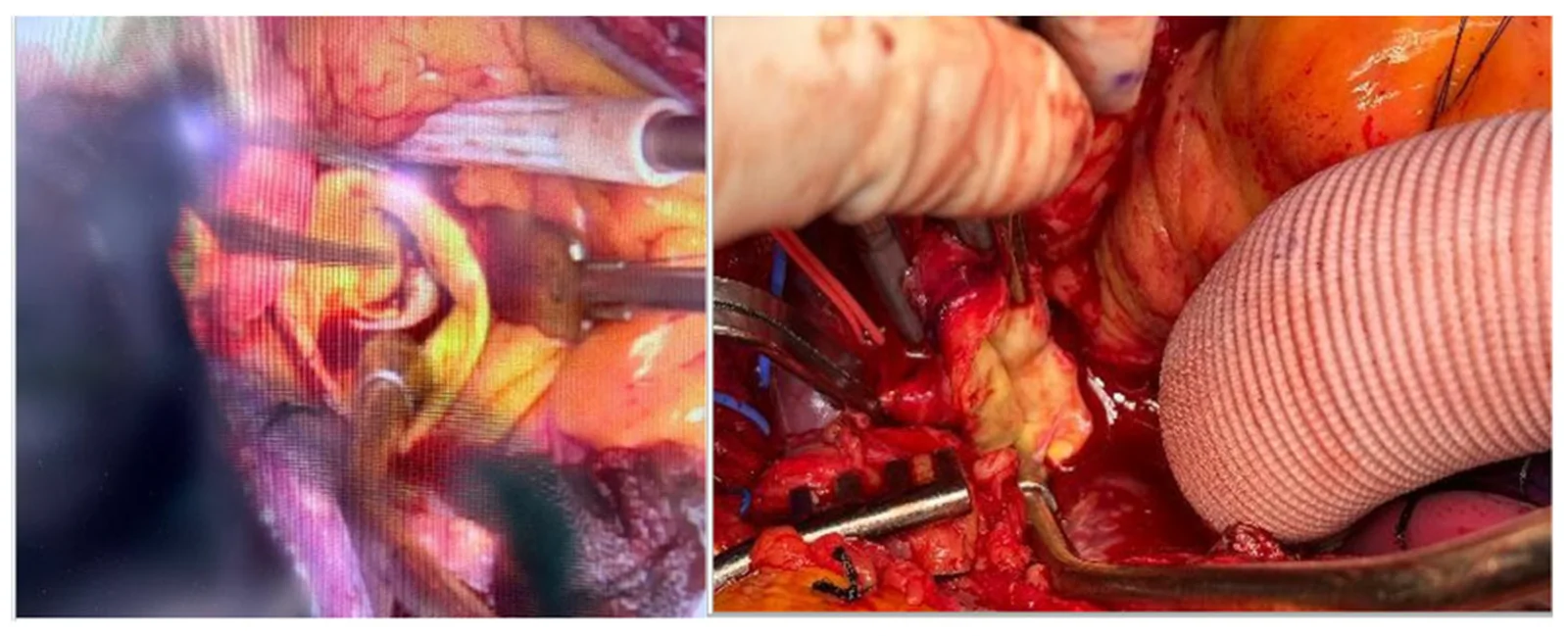
Intraoperative exploration of the ascending aorta. (**Left**) Direct visualization of the opened aortic root demonstrates smooth aortic intima and intact aortic valve leaflets (indicated by forceps) without evidence of an intimal tear or false lumen. (**Right**) Full-thickness inspection of the transected ascending aortic wall (held by forceps) alongside a prepared synthetic Dacron graft, confirming a normal aortic architecture without dissection or intramural hematoma.

**Figure 4:**
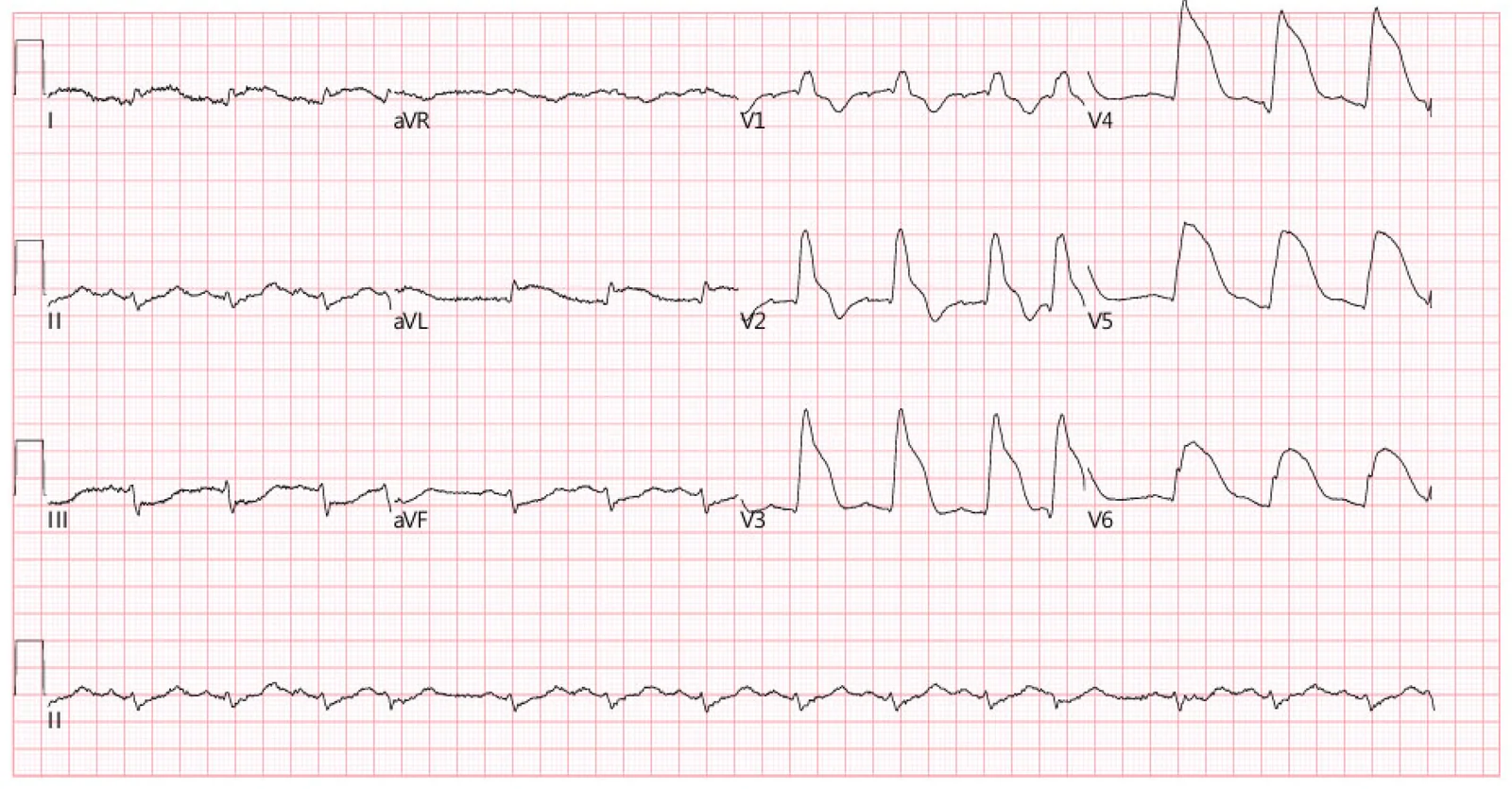
Electrocardiography revealed ST-segment elevation consistent with an acute anterior wall myocardial infarction (STEMI).

Preoperative transesophageal echocardiography (TEE) revealed a hyperechoic, flap-like structure in the aortic root (Figure 2). The combined CTA and TEE findings were considered sufficient in that the initial diagnosis of aortic dissection was established based on a consensus involving cardiology, cardiac surgery, radiology, and echocardiography experts. Although the cardiologist initially suspected acute myocardial infarction (AMI) and suggested coronary angiography, the initial electrocardiogram was atypical and of sub-optimal quality. Following a multidisciplinary discussion, the consensus ultimately aligned with the cardiac surgeon’s assessment—concluding that the patient most likely suffered cardiac arrest secondary to aortic dissection, requiring immediate surgical intervention. Intraoperatively, the heart appeared distended; however, no intimal tear or dissection was identified in the ascending aorta (Figure 3). On postoperative day one, electrocardiography revealed ST-segment elevation consistent with an acute anterior wall myocardial infarction (STEMI) (Figure 4). Emergent coronary angiography demonstrated two-vessel coronary artery disease, including 76% stenosis of the left anterior descending artery (LAD), 57% stenosis of the first diagonal branch (D1), and diffuse 51% stenosis of the proximal right coronary artery (RCA). A drug-eluting stent was deployed in the LAD, and balloon angioplasty was performed on D1.

**Figure 5:**
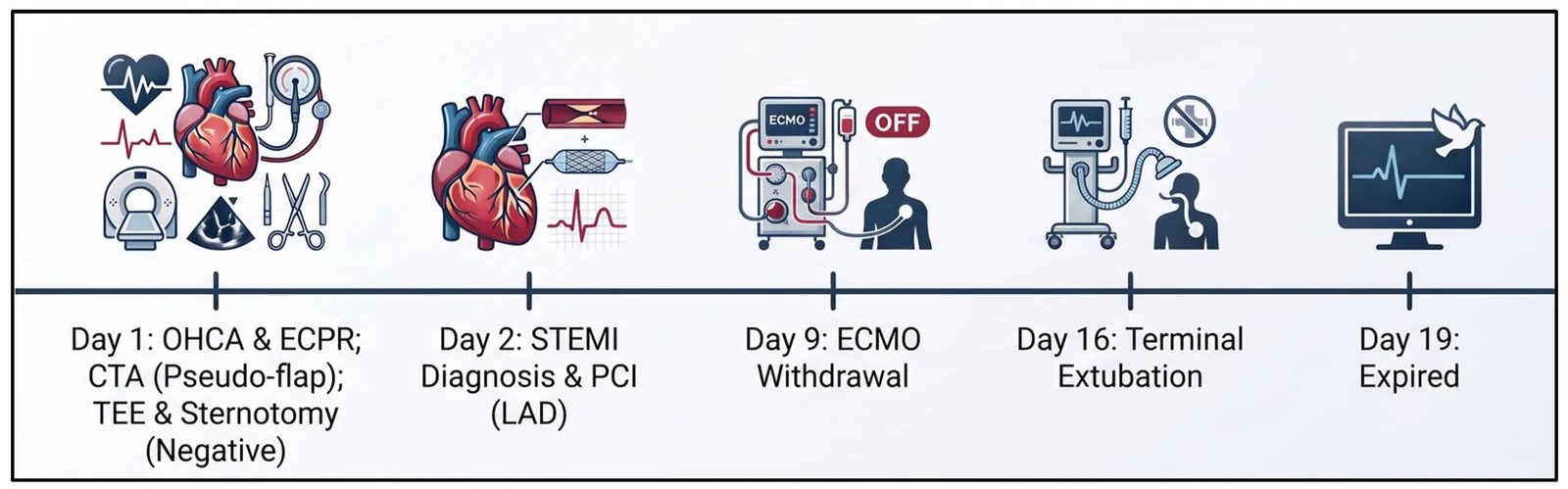
Clinical course and event timeline.

The clinical course was complicated by acute kidney injury requiring continuous renal replacement therapy and persistent severe LVEF depression (<20%). However, neurological evaluation later revealed irreversible hypoxic encephalopathy with diffuse cortical dysfunction. Due to this dismal neurological prognosis, the patient’s family elected for a do-not-resuscitate order. ECMO was palliatively discontinued on hospital day 9, after which a surprising recovery of LVEF to 68.6% was noted. Nevertheless, due to profound ongoing neurological impairment, compassionate terminal extubation was performed on day 16, and the patient expired on day 19 (Figure 5).

## 3. Discussion

This case illustrates a critical diagnostic pitfall in the emergency evaluation of patients undergoing extracorporeal cardiopulmonary resuscitation (ECPR): imaging findings cannot be interpreted in isolation from the underlying hemodynamic context. In patients receiving peripheral veno-arterial extracorporeal membrane oxygenation (VA-ECMO), profound alterations in aortic flow dynamics may substantially distort the appearance of both computed tomography angiography (CTA) and transesophageal echocardiography (TEE), generating findings that closely resemble life-threatening aortic pathology. This case underscores the necessity of context-aware imaging interpretation, in which imaging findings are systematically integrated with ECMO configuration, flow direction, and native cardiac output to guide high-stakes surgical decision-making during early emergency and post-resuscitation care.

### 3.1. Hemodynamic Mechanisms of Imaging Artifacts and Tailored CTA Strategies

The principal mechanism underlying this diagnostic pitfall is the profound alteration of aortic hemodynamics induced by peripheral VA-ECMO support [1]. Retrograde arterial inflow from the femoral cannula competes with antegrade native cardiac output, particularly in patients with severe cardiogenic shock or during ECPR [1].

The manifestations and precise locations of these flow disturbances are critically dependent on the level of native cardiac output. When native cardiac output is markedly reduced, laminar layers of unmixed blood typically form within the aortic root and ascending aorta [1]. The stark interface between unenhanced ECMO blood and contrast-enhanced native blood produces a contrast layering or “stagnant blood” artifact that closely mimics an intimal flap on CTA [2]. Conversely, when native cardiac output is partially preserved, the collision between retrograde ECMO flow and antegrade left ventricular output shifts the fluid dynamics, creating a volatile watershed zone—commonly within the thoracic aorta—where flow disturbances and imaging artifacts are most pronounced [3]. Consequently, these variable hemodynamic phenotypes significantly limit the diagnostic reliability of CTA in the VA-ECMO setting.

**Figure 6:**
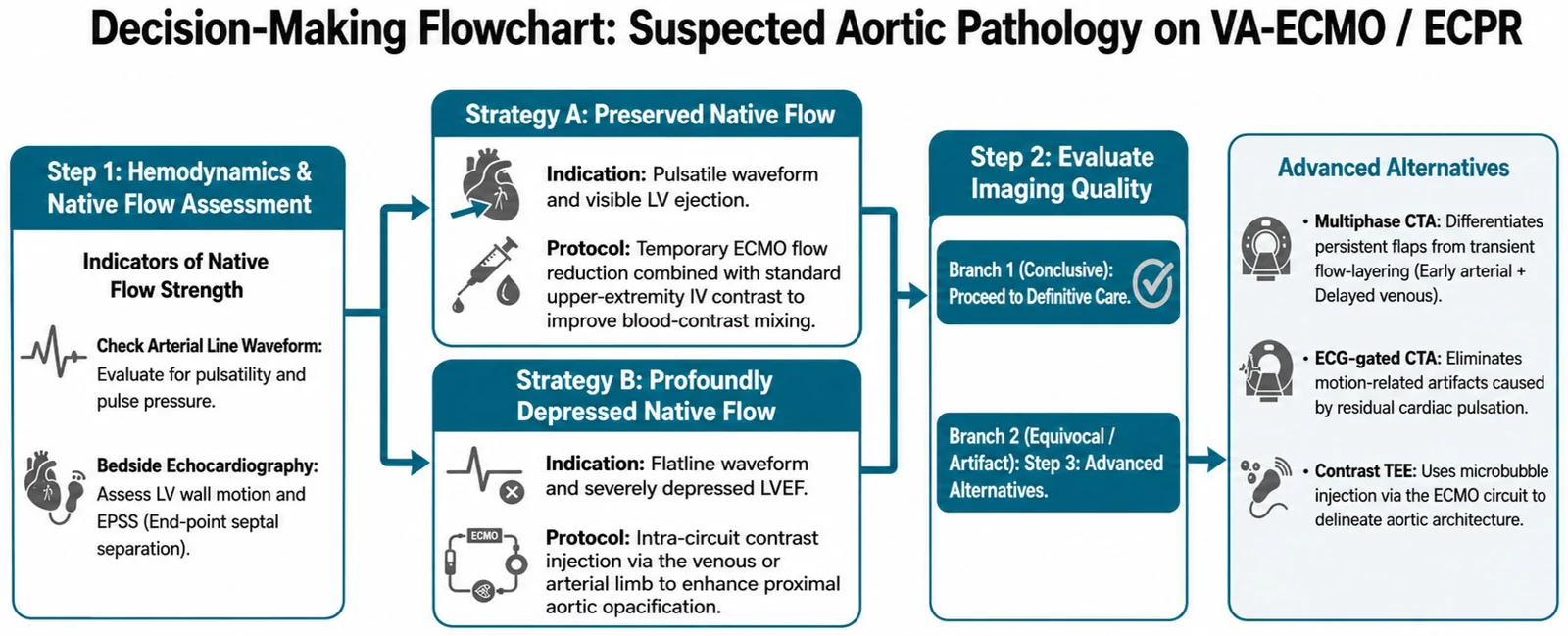
Diagnostic algorithm for acute aortic imaging under VA-ECMO. Native flow strength directs primary CTA protocol selection (Strategy A: flow reduction; Strategy B: intra-circuit contrast). Equivocal findings prompt advanced modalities (multiphase/ECG-gated CTA or contrast TEE) before surgical intervention.

To translate these hemodynamic principles into clinical practice and resolve the diagnostic dilemmas associated with ECMO support, we propose a practical diagnostic decision-making algorithm (Figure 6) for a tailored imaging strategy titrated to the estimated strength of the patient’s native cardiac output. In scenarios where native flow is relatively preserved—and the watershed zone is anticipated further downstream in the descending aorta [3]—a temporary reduction in ECMO flow rates combined with standard upper-extremity IV contrast administration can optimize blood–contrast mixing and minimize local flow interference (Strategy A, Figure 6) [2,4]. Conversely, in cases where native flow is profoundly depressed—shifting the predictable artifact zone proximally from the aortic arch to the ascending aorta [2]—administering contrast directly through the ECMO circuit (venous or arterial limb) is reported to enhance proximal aortic opacification (Strategy B, Figure 6) [4]. Furthermore, in either hemodynamic situation when optimal image quality is not achieved, alternative advanced approaches should be considered: a multiphase CTA protocol (early arterial and delayed venous phases) can differentiate persistent true intimal flaps from transient flow-layering artifacts [4], while an ECG-gated CTA can eliminate motion-related artifacts caused by residual cardiac pulsation [5].

### 3.2. TEE Utilization

TEE remains a widely utilized bedside modality for the evaluation of suspected aortic dissection, with reported diagnostic sensitivity approaching that of CT or MRI [6]. In select cases of cardiac arrest under ECMO support, TEE has successfully identified aortic dissection and guided cessation of ECMO therapy [7]. However, even expert TEE interpretation may be confounded under extracorporeal circulatory support, as altered flow dynamics can generate echocardiographic appearances indistinguishable from true intimal flaps. Recent reports by De Fazio et al. and Foster et al. describe cases in which ECMO- or bypass-related flow patterns produced pseudo-dissection findings on TEE, emphasizing the risk of false-positive diagnoses [8,9]. As a complementary technique, contrast-enhanced TEE using microbubble injection via the ECMO circuit has been proposed to delineate true luminal flow and cannula position, offering dynamic information beyond structural imaging alone [10]. This approach may be particularly useful in selected cases when computed tomography is unavailable, inconclusive, or potentially misleading under ECMO support.

In our case, a combination of CTA and TEE identified a suspicious lesion but failed to definitively demonstrate a true intimal tear. While urgent surgical repair of a true TAAD is life-saving, an unnecessary sternotomy confers prohibitive morbidity and mortality in an already critically ill ECPR patient [11]. This case highlights the critical role of multimodality imaging and underscores the necessity of interpreting imaging findings within the context of ECMO flow direction and native cardiac output to achieve an accurate diagnosis.

While these patient-specific imaging adaptations are highly plausible based on fluid dynamic principles, several limitations must be acknowledged. First, this physiology-based imaging strategy remains conceptual and is not yet supported by prospective validation. Second, clear-cut quantitative definitions to differentiate ‘strong’ versus ‘weak’ native flow in the hyperacute setting remain undefined. Furthermore, continuous real-time measurement of native cardiac output is impractical during CT scanning. Consequently, native flow strength must be inferred from clinical surrogates—such as pulse pressure, arterial line waveforms, and bedside echocardiography—assessed immediately prior to or following imaging. Third, the clinical efficacy and safety of adjusting ECMO flows or manipulating injection sites during acute resuscitation lack robust validation from large-scale prospective studies. Nonetheless, rather than relying solely on equivocal CTA findings obtained during standard protocols, this context-aware approach offers a physiological framework to prevent artifact-driven misdiagnoses. Future clinical trials and standardized institutional protocols integrating cross-disciplinary expertise are warranted to validate and refine these tailored imaging strategies in ECPR patients.

## 4. Conclusions

Evaluating suspected aortic dissection during ECPR poses a profound diagnostic challenge due to VA-ECMO-induced alterations in aortic flow dynamics, which can create catastrophic false-positive findings on standard imaging. Rather than relying strictly on conventional protocols, emergency physicians and intensivists should adopt a context-aware approach, integrating multi-modality imaging with the patient’s hemodynamic status. Crucially, tailor-made imaging strategies—such as adjusting ECMO flow for patients with preserved native output, or utilizing intra-circuit injections for those with profound cardiac depression—offer a physiological framework to prevent non-therapeutic, high-risk surgical interventions in these critically ill patients.

## Data Availability

The original contributions presented in this study are included in the article. Further inquiries can be directed to the corresponding author.

## Supplementary Materials

The following supporting information can be downloaded at: https://www.mdpi.com/article/10.3390/reports9030262/s1, Figure S1: Time–density curve and scan acquisition timing for the dual-phase chest CTA; Table S1: Institutional Criteria for ECPR Team Activation.
