# Supplementary material for: Aortic Dissection Mimicry Under Extracorporeal Membrane Oxygenation (ECMO) After Cardiac Arrest: A Case Report of Emergency Imaging Dilemmas
Source: Reports (MDPI). 2026 Aug 10;9(3):262. doi: 10.3390/reports9030262 (PMC13511023; doi:10.3390/reports9030262)
Supplement: Supplementary file 1 [file reports-09-00262-s001.zip › reports-4438876-supplementary.pdf]

## Supplementary file S1

Supplementary Table S1. Institutional Criteria for ECPR Team Activation

| Category | Criteria Details |
|---|---|
| **Inclusion Criteria**<br><br>*(MUST meet ALL conditions)* | • **Age:** Actual or estimated age between 18–70 years *(patients >70 years require attending physician evaluation)*.<br><br>• **Rhythm / Witness Status:** Witnessed collapse with immediate bystander CPR, **OR** an initial shockable rhythm (Ventricular Fibrillation [VF] or Pulseless Ventricular Tachycardia [pVT]).<br><br>• **Pre-hospital Transit Time:** Time elapsed from the emergency call (119) to arrival at the hospital ED is <30 minutes.<br><br>• **Etiology:** Non-traumatic cardiac arrest. |
| **Exclusion Criteria**<br><br>*(Do NOT activate if ANY apply)* | • **Baseline Functional Status:** Inability to perform activities of daily living (e.g., long-term care or nursing home residents).<br><br>• **Severe Comorbidities:** End-stage malignancy, chronic stroke with coma or paralysis, severe COPD, end-stage liver cirrhosis, or known history of AIDS.<br><br>• **Contraindications to Anticoagulation:** Severe trauma, suspected or confirmed aortic dissection, or acute intracranial hemorrhage.<br><br>• **Legal & Social Status:** Valid Do Not Resuscitate (DNR) status, or unaccompanied/homeless patients with no family present.<br><br>• **Futility:** Favorable neurological prognosis assessed as futile by the attending resuscitation physician. |

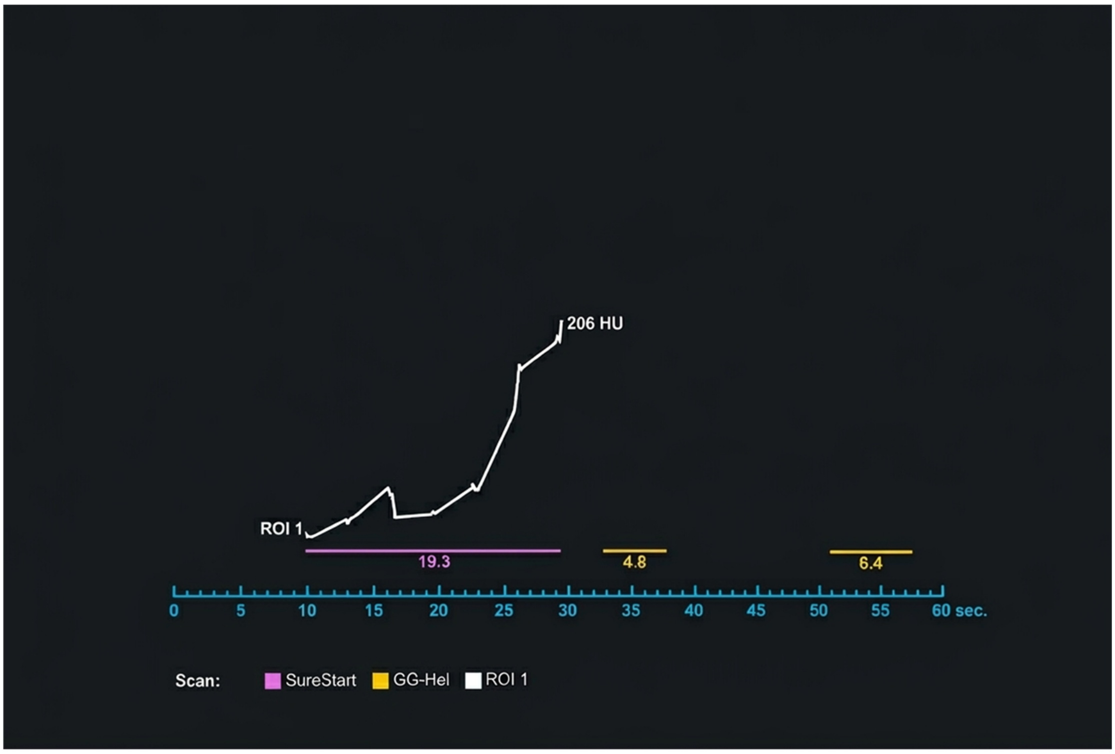


Supplementary Figure S1.

Time-density curve and scan acquisition timing for the dual-phase chest CTA. The horizontal axis represents elapsed time in seconds, with 0 seconds marking the onset of peripheral intravenous contrast administration. Bolus tracking was performed by placing a region of interest (ROI 1) in the aortic arch (pink bar, SureStart phase). Image acquisition was automatically triggered when attenuation exceeded the institutional threshold (>180 HU, reaching 206 HU at 29.5 seconds). The first scan phase (early arterial phase, indicated by the first yellow line at approximately 33–38 seconds) was performed immediately post-triggering. The second scan phase (indicated by the second yellow line at approximately 51–57 seconds) was initiated at a fixed delay of 45 seconds post-injection without dynamic ROI tracking. Because this second acquisition was performed at a predetermined time rather than tailored to delayed contrast wash-in dynamics, it may not represent a true delayed venous phase, thereby limiting its reliability in definitively differentiating true intimal flaps from flow-layering pseudo-flaps.
